# Physiological responses, body temperature, enteric methane and ruminal microbiota of lambs fed sugarcane silage and *Tithonia diversifolia*

**DOI:** 10.1007/s11250-026-05247-8

**Published:** 2026-07-27

**Authors:** Josiel Ferreira, Natana Mendes Marques, Mayara Isaias Vargas, Letícia Menezes dos Santos, Adibe Luiz Abdalla, Fernando Casanova Lugo, Ricardo Lopes Dias da Costa

**Affiliations:** 1 Instituto Federal de Educação, Ciência e Tecnologia Goiano, Campus Campos Belos, Campos Belos, GO 73840000 Brazil; 2 Centro de Pesquisa e Desenvolvimento em Zootecnia Diversificada, Instituto de Zootecnia/Apta/SAA-SP, Nova Odessa, SP 13380011 Brazil; 3 Laboratório de Nutrição Animal, Centro de Energia Nuclear na Agricultura, Universidade de São Paulo, Piracicaba, SP 13400970 Brazil; 4 Instituto Tecnológico de la Zona Maya, Tecnológico Nacional de México, Quintana Roo, México

**Keywords:** Greenhouse gases, *Ruminococcus flavefaciens*, Sheep, Thermography, Thermoregulation

## Abstract

This study evaluated the effects of replacing sugarcane silage with *Tithonia diversifolia* silage on physiological responses, body temperature, enteric methane (CH_4_) production, and selected ruminal microbial populations of lambs raised under tropical conditions. Twenty uncastrated crossbred lambs (½ Santa Inês × ½ Dorper), approximately 120 days old and with an initial body weight of 25.74 ± 2.47 kg, were assigned to two dietary treatments: exclusive sugarcane silage (SCA) or a 50:50 mixture of sugarcane silage and *T. diversifolia* silage (SCA + TD). Diets were formulated with a 60:40 forage-to-concentrate ratio. Physiological parameters, surface and infrared temperatures, enteric CH_4_ production, and ruminal microbial populations were evaluated. Data were analyzed using mixed models in R software. Lambs fed SCA showed greater respiratory rate (RR) and higher surface and infrared temperatures in most evaluated body regions compared with lambs fed SCA + TD (*p* < 0.05). Rectal temperature was not affected by dietary treatment (*p* > 0.05). No significant differences were observed for enteric CH_4_ production variables between treatments (*p* > 0.05). Dietary treatment affected *Ruminococcus flavefaciens* abundance, which was greater in lambs fed SCA + TD (*p* < 0.001), whereas the remaining ruminal microbial populations were not affected. The inclusion of *T. diversifolia* in sugarcane silage improved physiological and thermoregulatory responses of lambs and increased the abundance of a fibrolytic ruminal microorganism, although it did not reduce enteric CH_4_ production under the conditions evaluated in this study.

## Introduction

Sheep farming is a socioeconomically important activity for family-based production systems in several regions of the world. In Brazil, sheep production has considerable development potential due to the diversity of breeds adapted to different environmental and climatic conditions. However, seasonal fluctuations in forage availability in tropical regions remain one of the main constraints affecting animal productivity and increasing feeding costs in ruminant production systems (Carvalho et al. 2017; Wankhede et al. 2022).

Among the forage resources available in tropical regions, sugarcane (*Saccharum officinarum* L.) is widely used in ruminant feeding because of its high biomass yield, availability during the dry season, and relatively low production cost per unit of dry matter (Cabral et al. 2022). Nevertheless, sugarcane presents important nutritional limitations, including low crude protein concentration and reduced fiber digestibility associated with high levels of structural carbohydrates and lignification (Cabral et al. 2020; Oliveira et al. 2019; Bonfá et al. 2020). These characteristics may negatively affect ruminal fermentation efficiency and animal physiological responses, particularly under tropical environmental conditions.

In this context, *Tithonia diversifolia* (Hemsl.) A. Gray has emerged as a promising forage alternative for tropical ruminant systems due to its high productive potential and favorable nutritional composition (González-Sierra et al. 2019). The leaves of *T. diversifolia* contain elevated crude protein concentrations, ranging from 18.3 to 21.3%, in addition to secondary metabolites such as tannins, saponins, and phenolic compounds (Vargas Velázquez et al. 2022). These compounds have been associated with the modulation of ruminal fermentation and possible reductions in enteric methane (CH_4_) production through their effects on ruminal microbial populations, particularly methanogenic archaea and fibrolytic microorganisms (Cieslak et al. 2013).

Enteric CH_4_ emissions from ruminants represent an important environmental concern because livestock contributes substantially to global greenhouse gas emissions. Methane produced during ruminal fermentation is considered one of the main contributors to the environmental impact of livestock systems due to its high global warming potential compared with carbon dioxide (CO_2_) (Valencia Salazar et al. 2017; Khurana et al. 2023). Consequently, nutritional strategies capable of improving ruminal efficiency while mitigating CH_4_ emissions have received increasing scientific attention.

In addition to their effects on ruminal fermentation, dietary components may influence animal thermoregulation and physiological responses. Heat production associated with feed digestion, especially from fibrous diets, contributes to the animal’s thermal load and may intensify heat stress under tropical conditions (Ferreira et al. 2019, 2026; Barbosa et al. 2024). Physiological variables such as respiratory rate, heart rate, rectal temperature, and body surface temperature are important indicators of thermal stress and adaptive responses in ruminants. Infrared thermography has also been increasingly used as a non-invasive tool to evaluate body temperature distribution and thermal balance in livestock animals. Although previous studies have investigated the nutritional characteristics of *T. diversifolia* and its potential effects on ruminal fermentation, limited information is available regarding the integrated effects of replacing sugarcane silage with *T. diversifolia* on physiological responses, thermoregulation, enteric CH₄ production, and selected ruminal microbial populations in lambs raised under tropical conditions.

Therefore, we hypothesized that the partial replacement of sugarcane silage with *T. diversifolia* would improve physiological and thermoregulatory responses and modulate ruminal microbial populations, potentially influencing enteric CH_4_ production in lambs. Thus, the present study aimed to evaluate physiological parameters, body temperature, enteric methane production, and selected ruminal microbial populations of lambs fed sugarcane silage with or without the inclusion of *T. diversifolia*.

## Material and methods

### Ethics committee

The experiment was reviewed and approved by the Ethics Committee on the Use of Animals in Experimentation of the Institute of Animal Science (CEUA-IZ) under opinion number 346–2022.

### Experimental periods, location, and environmental characterization

This study was conducted in two experimental facilities. The first was at the Institute of Animal Science (IZ) of the São Paulo Agency for Agribusiness Technology (APTA), under the Secretariat of Agriculture and Supply, in Nova Odessa, São Paulo, Brazil, at geographic coordinates 22°46’39’’S, 47°17’45’’W. The lambs were housed in a confinement management barn for performance evaluation over a period of 68 days, including a 14-day adaptation period. During the performance trial, animals received the experimental diet and water ad libitum, and assessments of physiological parameters, thermographic images of body regions, and the first ruminal content collection were performed.

Subsequently, the experiment continued at the animal facilities of the Animal Nutrition Laboratory of the Center for Nuclear Energy in Agriculture at the University of São Paulo (LANA - CENA/USP) in Piracicaba, São Paulo, Brazil, at coordinates 22°23’31’’S, 47°38’57’’W. Here, the second ruminal content collection and in vivo evaluation of CH₄ production using gas collection chambers were conducted. Animals remained in the chambers for four days, including two days of adaptation and two days for in vivo CH_4_ measurements.

During the period in which animals were housed in the confinement barn, ambient temperature (AT, °C) and relative humidity (RH, %) were recorded using a meteorological station (WBGT Meter HMTGD-1800, Taiwan, China) at 08:00 h for thermal environment characterization. These measurements were used to calculate the Temperature-Humidity Index (THI; NRC, 1971) using the following equation:

$$\:\mathrm{T}\mathrm{H}\mathrm{I}=\mathrm{A}\mathrm{T}-[\left(0.31-0.31\times\:\mathrm{R}\mathrm{H}\right)\times\:\left(\mathrm{A}\mathrm{T}-14.4\right)]$$

Environmental characterization for the days corresponding to the measurement of physiological parameters (four collections) using AT, RH, and THI was separated and analyzed as a result, aiming to identify associations with the physiological variables.

### Animals and facilities

The experiment involved 20 male, intact lambs, crossbred ½ Santa Inês × ½ Dorper, approximately 120 days old, with an initial average body weight of 25.74 ± 2.47 kg. Prior to the experimental period, all animals were dewormed (Ripercol^®^, Zoetis, Campinas, SP, Brazil) and vaccinated with a multivalent clostridial vaccine (Poli Star^®^, Vallée, Montes Claros, MG, Brazil). Animals were identified with collars equipped with electronic chips fixed around the neck, which remained in place until the end of the experiment.

The confinement barn used during the performance trial consisted of two collective pens equipped with an automated system for individual feed and water intake measurements (Intergado Science^®^, Contagem, MG, Brazil). Each pen contained four electronic feeders and one drinker integrated with weighing sensors. The automated system was used exclusively for individual intake recording, whereas the experimental diets were manually supplied according to treatment.

Animals from both dietary treatments were equally distributed between the two collective pens, with five lambs from each treatment housed in each pen. This arrangement prevented confounding between pen and dietary treatment while allowing individual feed intake to be monitored through the automated feeding system. Therefore, the individual animal was considered the experimental unit for all statistical analyses.

Individual semi-open circuit gas collection chambers installed at the LANA – CENA/USP animal facilities were made of metal and polyethylene sealing plates, measuring 157 × 71 × 167 cm, with a volume of 1.9 m³, as described by Abdalla et al. (2008, 2012). Each chamber contained a feeder, a drinker, a fan, an exhaust system, and a digital thermometer to monitor internal temperature. In the barn, digital thermohygrometers (J-Prolab) were used to measure the ambient temperature outside the chambers, which averaged 26.78 °C, and wind speed (WS), which was 2.46 m/s², measured with a portable anemometer (Instrutherm AD-250).

### Treatments, feeding management and facility sanitation

The animals were fed either sugarcane silage or sugarcane silage combined with *T. diversifolia*, along with a concentrate (Sheep Feed for Confinement, Nutrição Animal Coopermota, Cândido Mota-SP, Brazil). For the preparation of *T. diversifolia* silage, plants were harvested at a height of 20–30 cm from the ground and chopped using a stationary chopper to achieve an average particle size of 1.0–1.5 cm. After harvesting of the *T. diversifolia*, the plant material was spread in a barn to dry for 12 h to reduce moisture content. For sugarcane silage preparation, 1% micro-processed lime was added to the fresh plant material prior to ensiling. The proportions of each treatment were weighed, mixed, and ensiled using a silage bagger in rectangular bags (200 μm, white), with a capacity of 20–40 kg. The diet was formulated with a 60:40 ratio of forage (sugarcane silage or sugarcane + *T. diversifolia* silage) to concentrate.

After the adaptation period, animals were randomly assigned to the two dietary treatments according to their initial body weight, resulting in 10 animals per treatment. Lambs from each treatment were then equally distributed between the two collective pens, ensuring that pen and dietary treatment were not confounded. The experimental diets consisted of either exclusive sugarcane silage (SCS) or a 50:50 (based on dry matter) mixture of sugarcane and *T. diversifolia* silage (SCS + TD). Diets were offered in two daily meals at 08:00 and 16:00 h during the performance trial and once daily (at 09:00 h) at the LANA facilities. Feeders and drinkers were cleaned daily in both experimental locations, and feed leftovers were weighed daily either via electronic feeders or manually. The proportion and chemical composition of the silages and concentrate are presented in Table 1.

**Table 1:** Chemical composition of experimental silages (treatments) and concentrate (% DM unless otherwise stated)

Variables (%)	Silage			Concentrate
	SCA	SCA + TD		
Dry matter (as-fed basis)	22.97	22.62		89.67
Crude protein	3.19	5.43		19.81
Non-fibrous carbohydrates	29.98	33.73		-
Ethereal extract	1.17	1.06		2.50
Mineral matter	6.23	6.17		5.14
Non-nitrogenous extractives	58.26	52.05		67.77
Acid detergent fiber	33.30	39.70		-
Neutral detergent fiber	55.68	57.49		-
Total digestible nutrients	62.62	59.73		75.13

Abbreviations: SCA = exclusive sugarcane silage; SCA + TD = mixture of 50% sugarcane silage and 50% *T. diversifolia* silage.

Analytical methods: Dry matter (Method No. 934.01), ether extract (Method No. 920.39), and mineral matter (Method No. 942.05) were determined according to AOAC (1990). Crude protein was determined using the Dumas method. Non-nitrogenous extractives and non-fibrous carbohydrates were determined according to AOAC (1995). Total digestible nutrients were estimated according to Kearl (1982)

During the performance trial, animals were housed in group pens with concrete floors and sugarcane bagasse bedding, which did not require replacement. In the LANA experiment, lambs were maintained in individual gas collection chambers. Before diet provision, the chambers were cleaned, removing feces, urine, and feed residues.

### Measurement of physiological parameters

Initially, animals were led in groups of 10 to a narrow corridor to perform the measurements, allowing 20 min for animal acclimation to reduce handling stress. The first variable measured was respiratory rate (RR, movements/min), determined by directly counting the movements of the left flank over 30 s without physical contact with the animals. The value was then multiplied by 2, as proposed by Gonçalves et al. (2023) and De Vasconcelos et al. (2023).

Heart rate (HR, bpm) was measured using a veterinary stethoscope (3 M™ Littmann^®^, Penlight; Shanghai, China) over 30 s, and the value was similarly multiplied by 2. Rectal temperature (RT, °C) was recorded with a digital clinical thermometer with a range up to 44 °C, inserted approximately 5 cm into the rectum, maintaining direct contact with the rectal mucosa until the sensor stabilized. Finally, body surface temperature (°C) of different body regions (head, muzzle, left flank, left hind hoof, and scrotum) was measured using a digital non-contact thermometer (Sem-Contato Premium) with a measurement range of 32–49 °C.

### Thermography

**Fig. 1:**
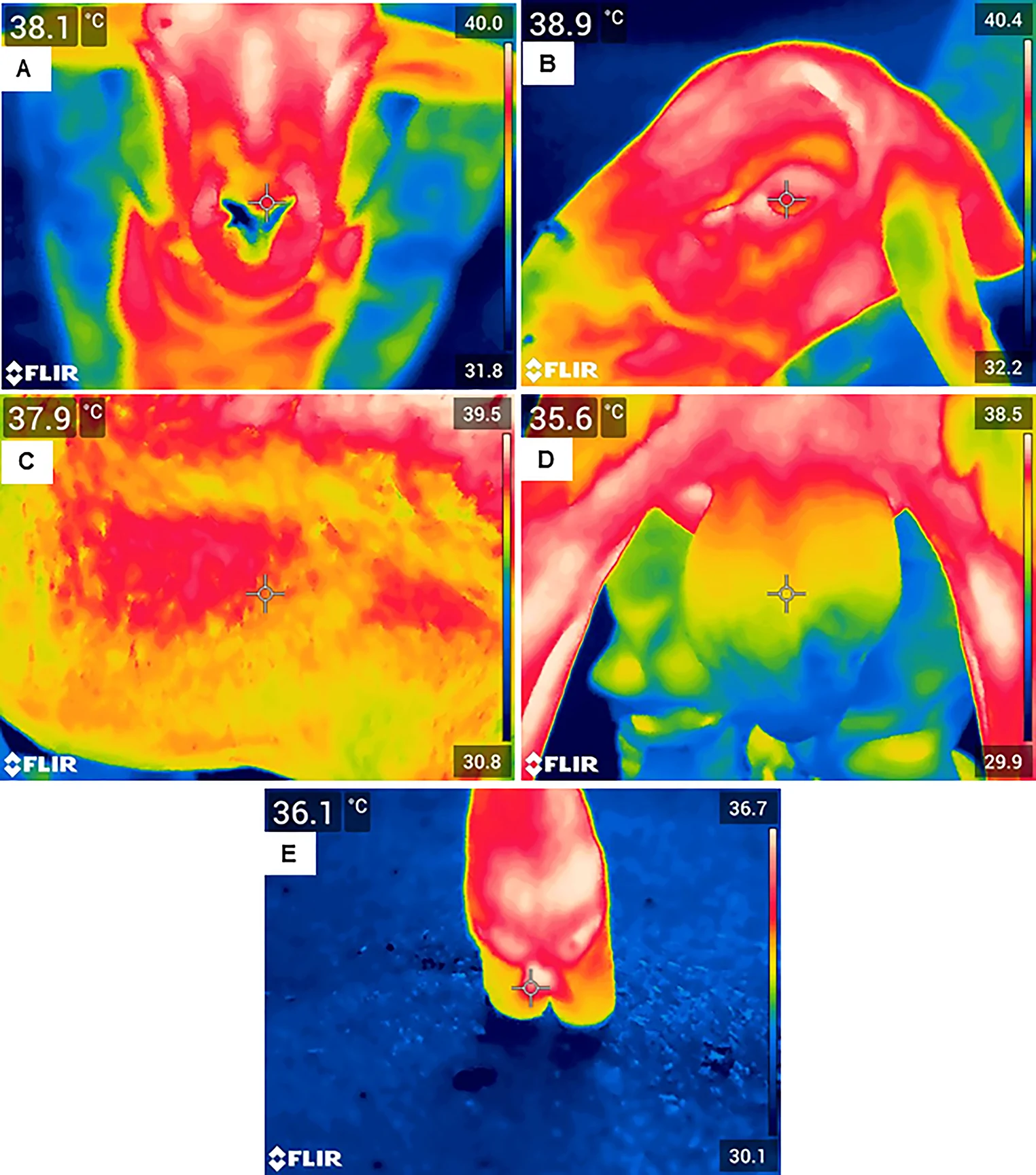
Infrared thermographic images of different body regions of lambs: (**A**) muzzle; (**B**) left eye; (**C**) left flank; (**D**) scrotum; and (**E**) hoof region

For thermographic imaging in the confinement barn, a FLIR thermographic camera (Flir E54, Flir Systems, Estonia) was used, with an infrared resolution of 320 × 240 pixels, sensitive to temperature variations of 0.5 °C, and a measurement range of -20 to 350 °C. The emissivity value was set at 0.98 for all thermographic images according to recommendations for biological tissues. The camera was calibrated following the manufacturer’s instructions before each collection period. Thermographic images were analyzed using FLIR Tools^®^ software (FLIR Systems, Estonia). Image acquisition was performed under shaded conditions to minimize interference from direct solar radiation and environmental reflections. For all collection periods, a standardized distance of 0.50 m from the animal was maintained to capture images according to the manufacturer’s recommendations. The selected body regions were: muzzle, left eye, left flank, scrotum, and right hind hoof (Fig. 1).

## Enteric methane quantification

In vivo CH_4_ production was quantified using semi-open circuit respiratory chambers, according to the methodology described by Abdalla et al. (2008, 2012). Animals remained in the chambers for four consecutive days, consisting of two days for adaptation to the chamber environment followed by two days of gas collection. Each collection period started at 09:00 h and ended at 08:00 h on the following day.

Methane production was continuously monitored for 23 h during each collection day, with one hour reserved for chamber cleaning and maintenance. Air was continuously removed from each chamber using an exhaust pump operating at an average airflow rate of 185 L/min, which was monitored using a digital anemometer (AD-250 Digital, Instrutherm, São Paulo, Brazil).

During gas collection, internal air temperature (AT_i_, °C), relative humidity (RH_i_, %), and internal wind speed (WS_i_, m/s) were recorded inside each chamber at 08:00, 11:00, 14:00, and 17:00 h. Air samples from each chamber were collected into polyethylene balloons connected to the exhaust system. At the end of each collection period, gas samples were homogenized, and 10 mL aliquots were collected in triplicate using 20 mL syringes (Becton-Dickson Surgical Industry, Curitiba, Brazil) and transferred into 10 mL vacuum-sealed tubes for CH_4_ analysis.

Methane concentration was determined using a gas chromatograph (Shimadzu GC2014, SINC Brasil, São Paulo, Brazil) equipped with a flame ionization detector (FID) and a micro-packed Shincarbon ST 100/120 column (1 m × 1.587 mm × 1.0 mm; Restek, Bellefonte, PA, USA). Quantification was performed using a calibration curve prepared with commercial methane standards at concentrations of 0, 30, 90, and 120 mL/L (White Martins PRAXAIR Industrial Gases Inc., Osasco, SP, Brazil; 995 mL/L purity).

Methane production was expressed as liters per day (L/d), liters per day per metabolic body weight (L/d/BW⁰·⁷⁵), and liters per day per dry matter intake (L/d/DMI).

## Ruminal content collection

Ruminal content samples were collected from each animal prior to the first feeding of the day using a silicone esophageal probe connected to a syringe via suction. After collection, samples were stored in 1.5 mL microtubes and maintained at − 80 °C. DNA extraction from each ruminal content sample was performed using the QIAamp^®^ Fast Stool Mini Kit (Qiagen, Valencia, USA) according to the “DNA Isolation from Feces for Pathogen Detection” protocol recommended by the manufacturer. DNA concentration (ng/µL) and purity (260/280 ratio) were determined using a BioDrop µLITE spectrophotometer (Biochrom, Cambridge, UK). For analyses, the final DNA concentration of 10 ng/µL was standardized for all samples.

To identify and quantify microorganisms, DNA samples from each animal were subjected to qPCR reactions using species-specific target genes and primer sets previously described by Correa et al. (2021) and Ovani et al. (2026). qPCR assays were conducted on a CFX96 Real-Time PCR system (Bio-Rad) with a final reaction volume of 10 µL. Briefly, each reaction contained 0.3 µL of each primer (10 µM stock solution), 2 µL of 5× HOT FIREPol EvaGreen qPCR Mix Plus (Solis BioDyne, Tartu, Estonia), 5.4 µL of ultrapure water, and 2 µL of DNA (10 ng/µL). Thermal cycling conditions were: enzyme activation at 95 °C for 15 min, followed by 35 cycles of denaturation at 95 °C for 15 s, annealing at 60 °C for 20 s, and extension at 72 °C for 20 s. After amplification, a dissociation curve was generated in a single cycle from 65 to 95 °C, with increments of 0.5 °C and fluorescence detection every 5 s.

Results were analyzed based on the quantification cycle (Cq) values recorded at the end of each reaction. The threshold line was set at 100 RFU (relative fluorescence units, log scale) for all qPCR assays. All samples and controls were run in duplicate, and samples with a standard deviation of the Cq > 0.5 were reanalyzed.

For each microorganism, a calibration curve was constructed using serial dilutions of purified PCR products adjusted to an initial concentration of 10 ng/µL. The initial quantity used for the calibration curve was based on DNA copy number (CN) according to the formula described by Ke et al. (2006):

$$\:\mathrm{N}\mathrm{u}\mathrm{m}\mathrm{b}\mathrm{e}\mathrm{r}\:\mathrm{o}\mathrm{f}\:\mathrm{c}\mathrm{o}\mathrm{p}\mathrm{i}\mathrm{e}\mathrm{s}\:/\:{\upmu\:}\mathrm{L}=\:\frac{\begin{aligned}&{6.022\:\times\:10}^{23}(\mathrm{c}\mathrm{o}\mathrm{p}\mathrm{i}\mathrm{e}\mathrm{s}/\mathrm{m}\mathrm{o}\mathrm{l})\cr&\times\:\mathrm{c}\mathrm{o}\mathrm{n}\mathrm{c}\mathrm{e}\mathrm{n}\mathrm{t}\mathrm{r}\mathrm{a}\mathrm{t}\mathrm{i}\mathrm{o}\mathrm{n}\:(\mathrm{g}/{\upmu\:}\mathrm{L})\end{aligned}}{\mathrm{M}\mathrm{o}\mathrm{l}\mathrm{e}\mathrm{c}\mathrm{u}\mathrm{l}\mathrm{a}\mathrm{r}\:\mathrm{w}\mathrm{e}\mathrm{i}\mathrm{g}\mathrm{h}\mathrm{t}\:(\mathrm{g}/{\upmu\:}\mathrm{L})}$$

where:

6.022 × 10^23^ is Avogadro’s number;

Molecular weight is the double-stranded nucleotide molecule value (330 × 2) multiplied by the fragment size (PCR product size for each microorganism).

Reaction efficiency (E) for each qPCR assay was determined using:

$$\%E = [10^{(-1/slope)}-1] \times 100$$

where the slope refers to the tangent line of the calibration curve ( Pfaffl and Hageleit 2001; Vandesompele et al. 2009).

The detection limit (DL) for each qPCR assay/microorganism was defined as the last DNA dilution exhibiting ≥ 80% amplification based on Cq values. Specificity was determined from amplification results and dissociation curve profiles.

### Experimental design and statistical analysis

A completely randomized design was used, with two dietary treatments and 10 animals per treatment. The experimental unit was the individual animal.

Data were analyzed using mixed models implemented in R software (Core Team 2021) using the *lme4* (Bates et al. 2015), *lmerTest* (Kuznetsova et al. 2017), and *emmeans* (Lenth 2024) packages. Dietary treatment, collection period, and their interaction were included as fixed effects, whereas animal was considered a random effect. Repeated measurements over time were accounted for by modeling collection period within animal.

The statistical model was represented as:

$$\:{Y}_{ijk}=\mu\:+{T}_{i}+{C}_{j}+{{\left(T\times\:C\right)}_{ij}+A}_{k}+{\varepsilon}_{ijk}$$

where $\:{Y}_{ijk}$ is the observed value; µ\muµ is the overall mean; $\:{T}_{i}$ is the fixed effect of dietary treatment; $\:{C}_{j}$ is the fixed effect of collection period; $\:{\left(T\times\:C\right)}_{ij}$ is the interaction between treatment and collection period; $\:{A}_{k}$ is the random effect of animal; and $\:{\varepsilon }_{ijk}$ is the residual error.

Normality of residuals and homogeneity of variances were evaluated using the Shapiro–Wilk and Levene tests, respectively. When significant effects were detected, least-square means were compared at a 5% significance level.

Pearson correlation analyses were performed to evaluate associations among physiological parameters, body surface and infrared temperatures, environmental variables, enteric methane production, and ruminal microbiota. Only statistically significant correlations (*p* < 0.05) with correlation coefficients ≥ 0.50 were considered biologically relevant and discussed.

## Results

**Fig. 2:**
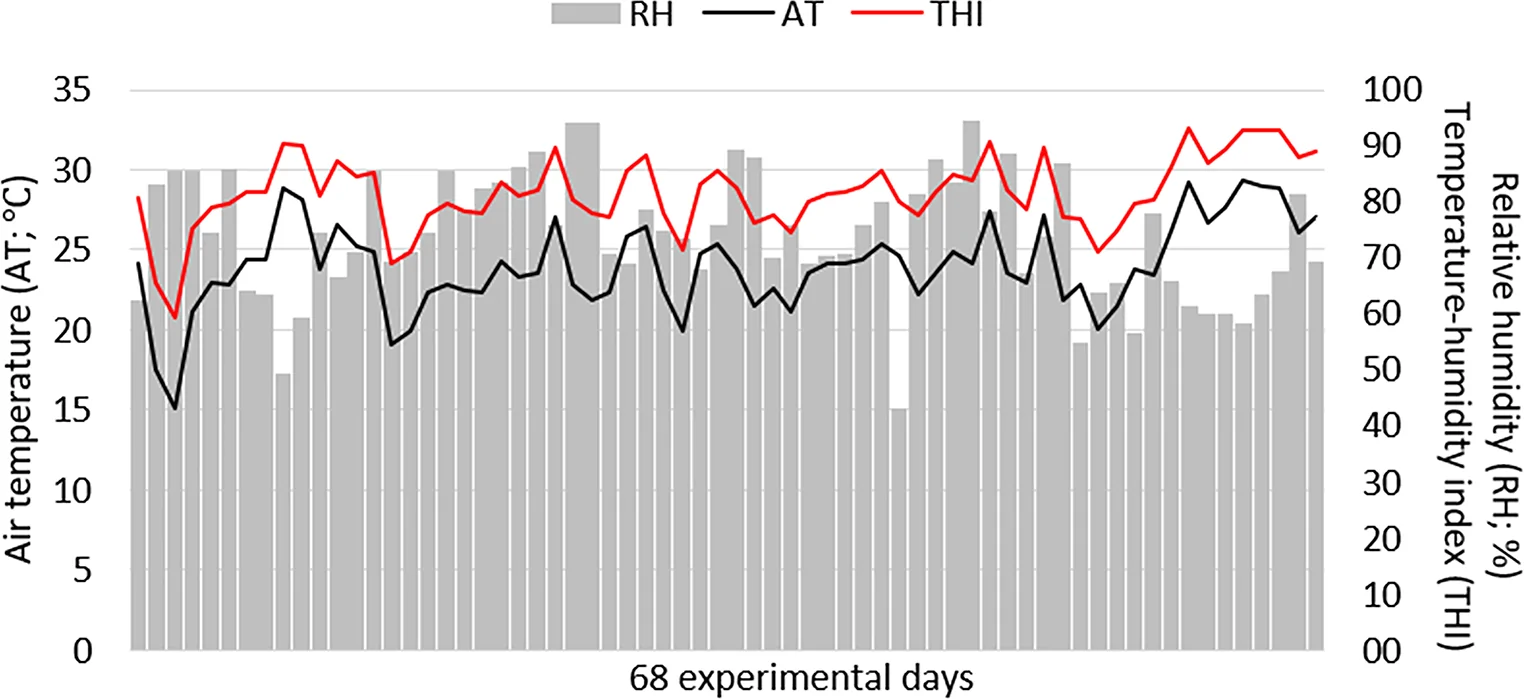
Variation in ambient temperature (AT), relative humidity (RH), and temperature-humidity index (THI) throughout the experimental period

Environmental conditions recorded during the experimental period are presented in Fig. 2. During this period, the maximum ambient temperature (AT) and relative humidity (RH) were 29.03 °C and 94.6%, respectively, whereas the minimum values were 15.1 °C and 43.3%, respectively. These environmental conditions resulted in a temperature-humidity index (THI) of 81.5, indicating conditions of environmental heat stress.

**Fig. 3:**
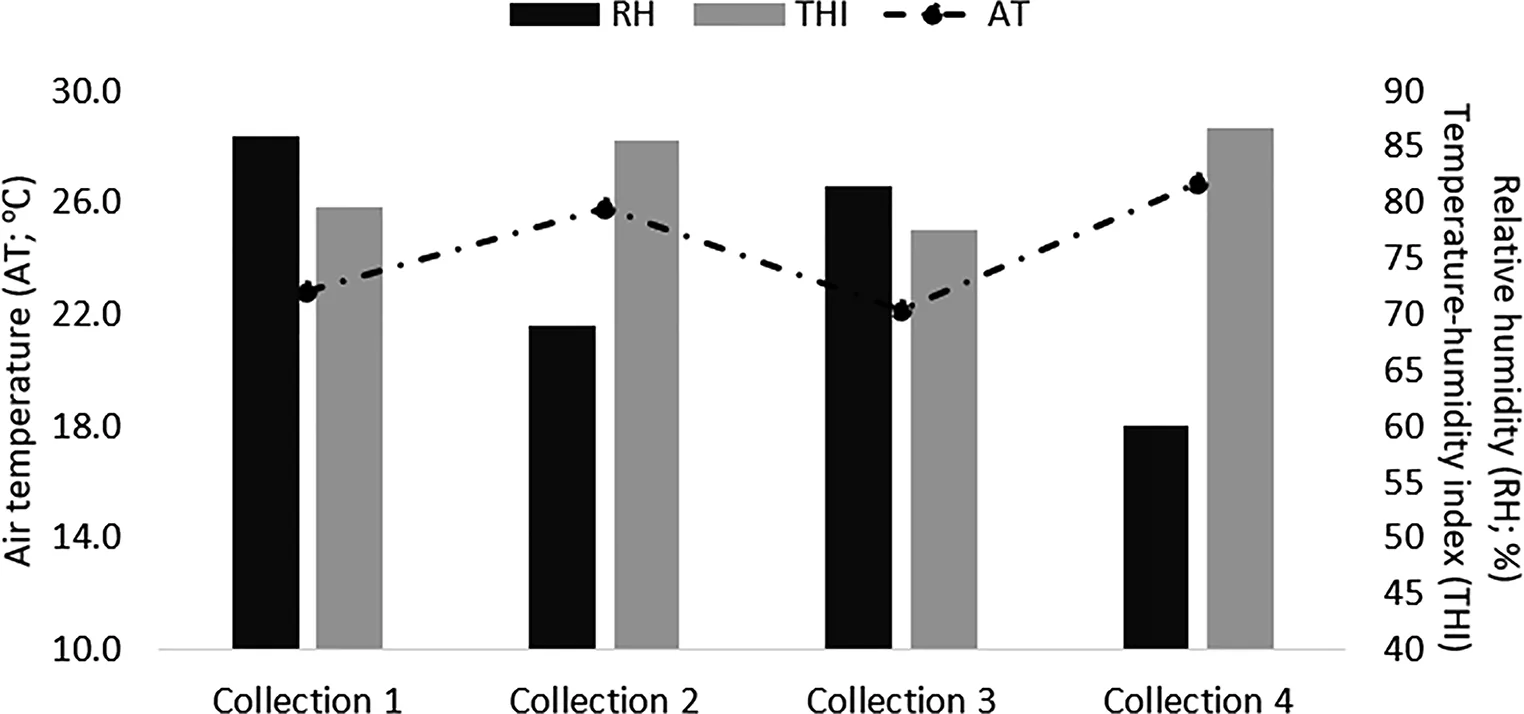
Ambient temperature (AT), relative humidity (RH), and temperature-humidity index (THI) during the collection periods for physiological and body temperature measurements

The measurements of AT and RH, as well as THI values obtained during the four collection periods corresponding to physiological and body temperature evaluations, are presented in Fig. 3. Collections 2 and 4 showed the highest AT and lowest RH, which resulted in higher THI values. Table 2 shows the overall mean (± standard deviation), minimum, and maximum of the environmental variables and THI across the four collections. AT varied by 4.50 °C and RH by 25.89% between minimum and maximum values, while THI varied by 9.13.

**Table 2:** Environmental variables and temperature-humidity index (THI) during the experimental period

Traits	Unit	Mean	Standard deviation	Minimum	Maximum
Air temperature	°C	24.3	2.2	22.2	26.7
Relative humidity	%	75.3	11.8	60.0	85.9
Temperature-humidity index	-	82.6	4.5	77.7	86.8

The physiological parameters of the animals fed different diets are presented in Table 3. No treatment × collection interaction was observed (*p* > 0.05) was detected for any variable. The RR and HR differed between treatments, with animals fed exclusively sugarcane silage (SCA) showing higher RR and lower HR. No differences (*p* > 0.05) were observed for RT between treatments.

**Table 3:** Least-square means ± SEM of physiological parameters of lambs fed sugarcane silage (SCA) or sugarcane with *T. diversifolia* (SCA + TD)

Traits	Unit	Treatments		*p*-value
		SCA	SCA + TD	
Respiratory rate	movements/min	49.3 ± 2.6	41.6 ± 2.0	0.022
Heart rate	bpm	82.6 ± 1.8	89.8 ± 2.5	0.020
Rectal temperature	°C	38.9 ± 0.1	38.9 ± 0.1	0.824

Abbreviations: SCA = exclusive sugarcane silage; SCA + TD = mixture of 50% sugarcane silage and 50% *T. diversifolia* silage

Table 4 presents the least-square means of surface temperatures (ST) and infrared temperatures (IT) for different body regions of the lambs. No interaction between treatments and collections was observed (*p* > 0.05) for either group of variables. Animals fed SCA showed higher surface temperatures in all evaluated regions (*p* < 0.05), with the flank surface temperature (FSt) showing the greatest difference (1.43 °C) and scrotal surface temperature (SSt) the smallest (1.23 °C). All IT values differed between treatments (*p* < 0.05), except for the infrared eye temperature (ITe). Higher temperatures were observed in the body regions (muzzle, flank, scrotum, and leg) of animals fed SCA.

**Table 4:** Least-square means ± SEM of surface and infrared temperatures (°C) of different body regions of lambs fed sugarcane silage (SCA) or sugarcane with *T. diversifolia* (SCA + TD)

Traits	Treatments		*p*-value
	SCA	SCA + TD	
Head surface temperature	32.65 ± 0.31	31.38 ± 0.26	0.002
Nasal surface temperature	31.17 ± 0.26	29.85 ± 0.28	< 0.001
Flank surface temperature	32.50 ± 0.41	31.07 ± 0.32	< 0.001
Posterior region of the hoof temperature	27.85 ± 0.32	26.49 ± 0.29	0.002
Scrotal surface temperature	31.63 ± 0.21	30.40 ± 0.25	< 0.001
Infrared eye temperature	37.14 ± 0.21	37.07 ± 0.18	0.788
Infrared muzzle temperature	33.32 ± 0.40	31.90 ± 0.51	0.031
Infrared flank temperature	34.26 ± 0.43	32.55 ± 0.36	0.003
Infrared hoof temperature	30.90 ± 0.45	29.17 ± 0.48	0.022
Infrared scrotal temperature	34.28 ± 0.19	33.25 ± 0.19	< 0.001

The least-square means of environmental variables and THI within the respirometry chambers are shown in Table 5. AT varied by 7.98 °C, RH by 38.4%, and WS by 1.32 m/s^2^ between minimum and maximum values, while THI varied by 14.36. The thermal environment and thermal comfort index for the respirometry chambers indicated more stressful conditions than in the confinement barn.

**Table 5:** Environmental variables and temperature-humidity index (THI) inside the respirometric chambers during the experimental period

Traits	Unit	Mean	Standard deviation	Minimum	Maximum
Air temperature	°C	27.8	1.9	25.7	33.7
Relative humidity	%	77.5	9.09	47.0	85.4
Wind speed	m/s	1.61	0.31	0.9	2.28
THI	-	81.5	3.3	77.8	92.2

The least-square means of in vivo CH_4_ production are presented in Table 6. No significant differences were observed for CH_4_ production variables between treatments.

**Table 6:** Least-square means ± SEM of enteric methane (CH_4_) production of lambs fed sugarcane silage (SCA) or mixture of sugarcane with *T. diversifolia* (SCA + TD)

Trait	Unit	Treatments		*p*-value
		SCA	SCA + TD	
CH_4_	L/d	9.53 ± 0.56	11.27 ± 0.71	0.180
CH_4_	L/kg BW^0,75^	0.72 ± 0.04	0.83 ± 0.06	0.264
CH_4_	L/Kg DMI/d	9.17 ± 0.56	9.40 ± 0.74	0.860

Abbreviations: BW^0.75^ = metabolic body weight; DMI = dry matter intake

The total populations of major ruminal microorganisms for both treatments are presented in Table 7. Dietary treatment affected *Ruminococcus flavefaciens* abundance, which was greater in lambs fed SCA + TD compared with SCA (*p* < 0.001). No significant effects of dietary treatment were observed for the remaining ruminal microorganisms evaluated (*p* > 0.05). In addition, no effects of collection period or treatment × collection interaction were detected for ruminal microbial populations.

**Table 7:** Least-square means ± SEM of ruminal microbial populations of lambs fed sugarcane silage (SCA) or mixture of sugarcane with *T. diversifolia* (SCA + TD)

Microorganisms	Treatments		*p*-value
	SCA	SCA + TD	
Total bacteria	5.03 ± 0.06	4.82 ± 0.06	0.073
Total fungi	3.99 ± 0.11	4.20 ± 0.09	0.295
Protozoa	5.95 ± 0.11	6.33 ± 0.10	0.074
Methyl coenzyme M reductase A	3.68 ± 0.13	3.50 ± 0.14	0.515
*Ruminoccooccus albus*	3.78 ± 0.03	3.96 ± 0.15	0.531
*Selenomonas ruminantium*	2.74 ± 0.08	2.76 ± 0.10	0.919
*Fibrobacter succinogenes*	4.80 ± 0.07	4.78 ± 0.06	0.880
*Ruminococcus flavefaciens*	4.18 ± 0.07	4.68 ± 0.05	< 0.001

Abbreviations: Values are expressed as log_10_ DNA copy number per ng/µL of DNA, except protozoa, which are expressed per mL of ruminal content

**Fig. 4:**
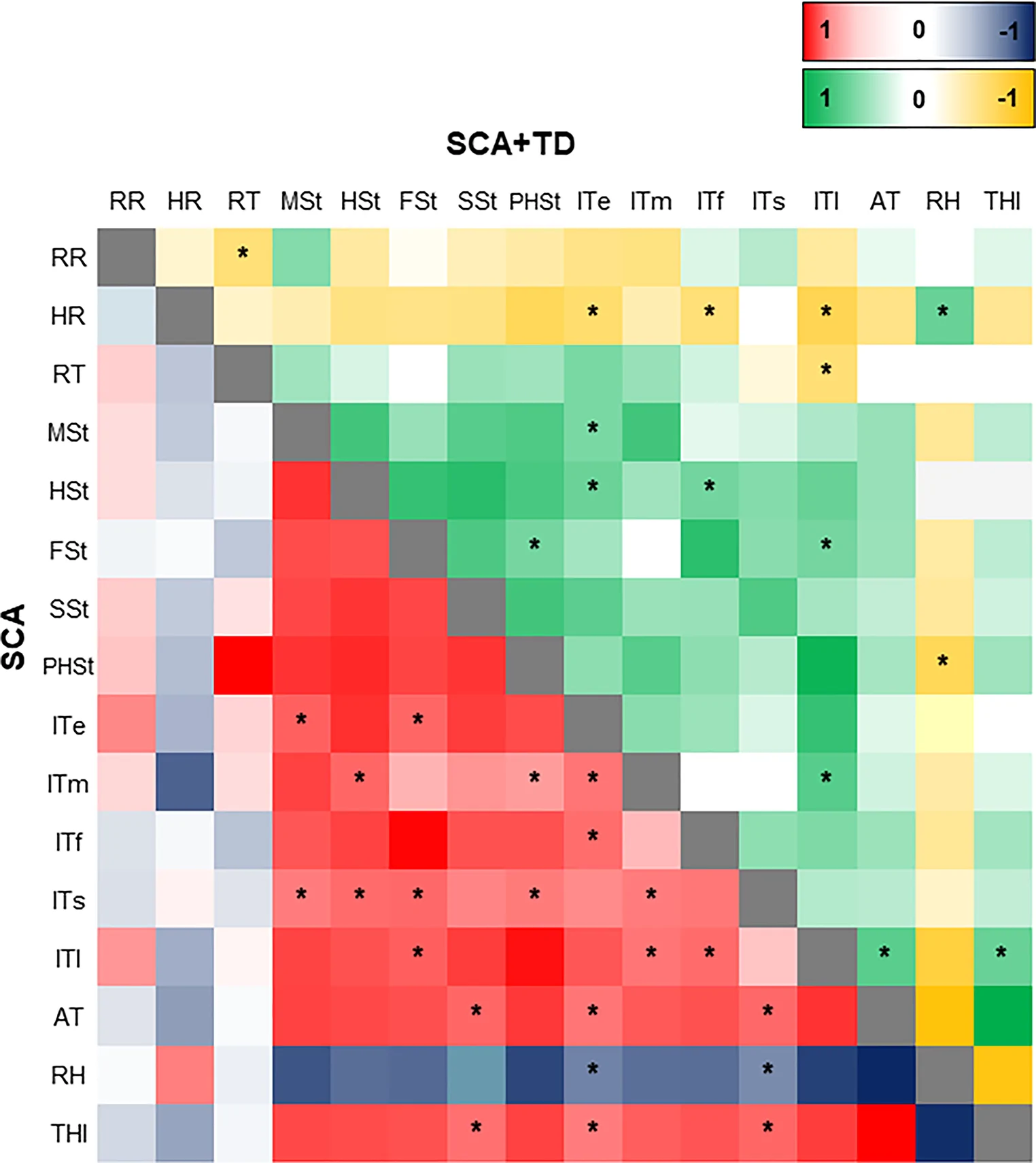
Heatmap of Pearson correlation coefficients among physiological parameters, body surface and infrared temperatures, and environmental variables of lambs fed sugarcane silage (SCA) or sugarcane silage with *T. diversifolia* (SCA + TD). Abbreviations: RR, respiratory rate; HR, heart rate; RT, rectal temperature; MSt, muzzle surface temperature; HSt, head surface temperature; FSt, flank surface temperature; SSt, scrotal surface temperature; HfSt, hoof surface temperature; ITe, infrared eye temperature; ITm, infrared muzzle temperature; ITf, infrared flank temperature; ITs, infrared scrotal temperature; ITh, infrared hoof temperature; AT, air temperature; RH, relative humidity; THI, temperature-humidity index. Note: Significant correlations (*p* ≤ 0.05) are indicated by *. Positive and negative correlations indicate the direction and strength of associations between variables

Figure 4 illustrates the Pearson correlations among physiological parameters, body surface and infrared temperatures, and environmental variables for both dietary treatments. In the SCA treatment, physiological variables showed no significant correlations with environmental parameters (*p* > 0.05). In contrast, body temperature variables were more strongly associated with environmental conditions, particularly AT and THI. Positive correlations were observed between AT and SSt, ITe, and ITs, whereas RH was negatively associated with infrared temperature variables. Similarly, increases in THI were positively correlated with SSt, ITe, and ITs.

For lambs fed SCA + TD, fewer significant correlations were observed among body temperature variables compared with the SCA treatment. The RR was negatively correlated with RT, while HR showed negative associations with infrared temperatures and a positive association with RH. Positive correlations were also observed among infrared temperature variables, particularly involving ITl. Environmental variables, especially AT and THI, were positively associated with ITl in the SCA + TD treatment.

**Fig. 5:**
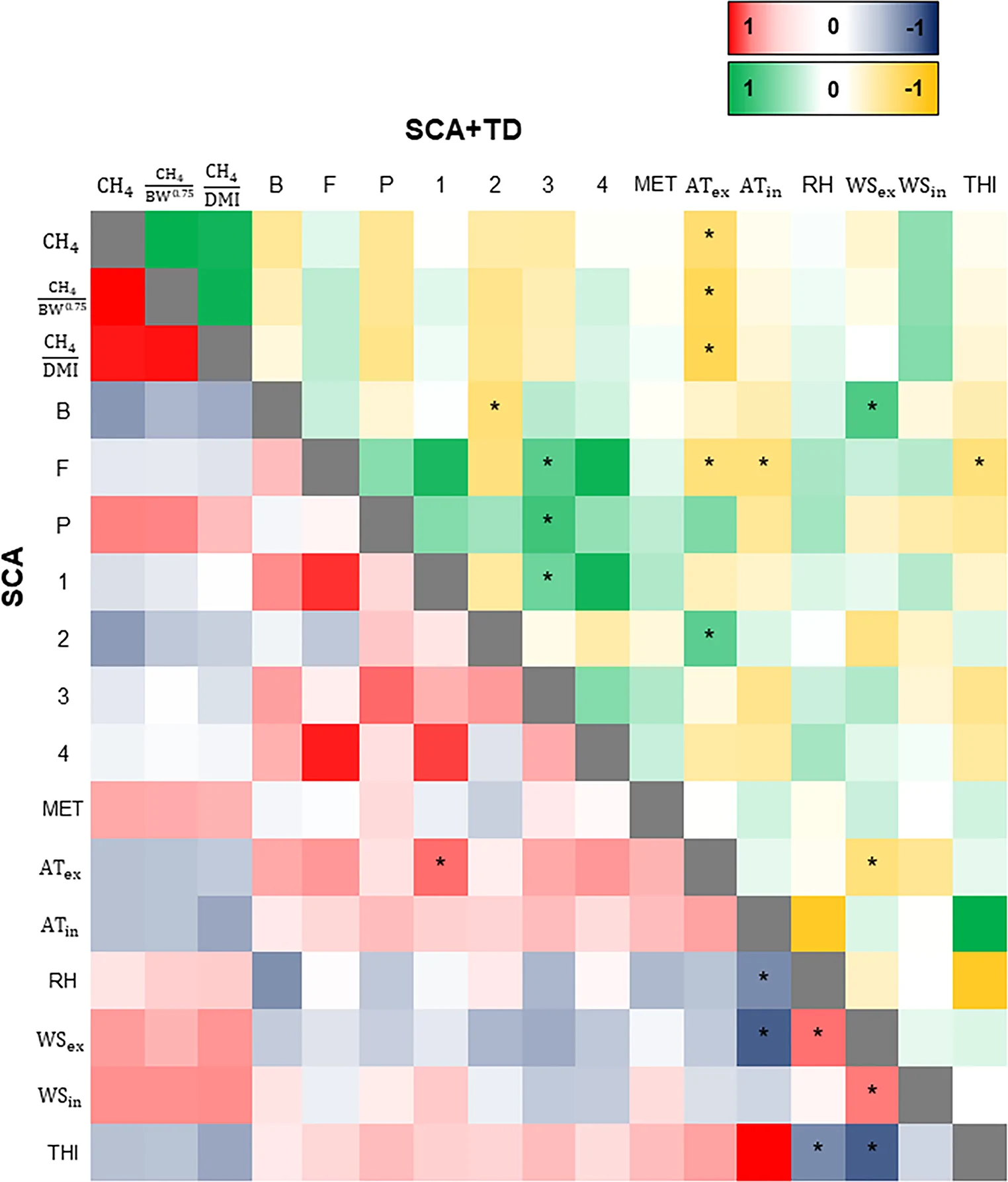
Heatmap of Pearson correlation coefficients among enteric methane production, ruminal microbial populations, and environmental variables of lambs fed sugarcane silage (SCA) or sugarcane silage with *T. diversifolia* (SCA + TD). Abbreviations: CH_4_, methane production; CH_4_/BW^0.75^, methane production per metabolic body weight; CH_4_/DMI, methane production per dry matter intake; B, total bacteria; F, total fungi; P, protozoa; *Ruminococcus albus*; *Selenomonas ruminantium*; *Fibrobacter succinogenes*; *Ruminococcus flavefaciens*; MET, methyl coenzyme M reductase A (mcrA) gene; ATex, external air temperature; ATin, internal air temperature; RH, relative humidity; WSex, external wind speed; WSin, internal wind speed; THI, temperature-humidity index. Note: Significant correlations (*p* ≤ 0.05) are indicated by *. Positive and negative correlations indicate the direction and strength of associations between variables

Figure 5 presents the correlations among CH_4_ production, ruminal microbial populations, and environmental variables in the respirometry chambers. In the SCA treatment, CH₄ production variables showed no significant associations with the evaluated ruminal microorganisms. Environmental variables were primarily correlated among themselves, while increases in ATex were positively associated with *Ruminococcus albus* abundance.

In the SCA + TD treatment, reductions in ATex were associated with lower CH_4_ production, CH_4_/BW^0.75^, and CH_4_/DMI values. Environmental conditions were also associated with changes in microbial populations, particularly fungi and *Selenomonas ruminantium*. Positive correlations were observed between *Fibrobacter succinogenes* and fungi, protozoa, and *R. albus*. Overall, environmental variables showed stronger associations with methane production and microbial populations in the SCA + TD treatment than in the SCA treatment.

## Discussion

Feed intake and physiological responses in ruminants are strongly influenced by environmental conditions and diet composition, particularly under tropical production systems (Ferreira et al. 2019; Tüfekci and Sejian 2023). Dietary characteristics such as fiber concentration and crude protein content can alter ruminal fermentation patterns, metabolic heat production, and thermoregulatory responses, potentially affecting animal performance and welfare (Pereira et al. 2021; Silva et al. 2021).

In the present study, RR and HR were above the physiological reference ranges reported for sheep, whereas RT remained within the normal range for the species (Reece, 2020). These responses were likely associated with the environmental conditions observed during the experimental period, particularly the elevated THI, which indicated thermal stress conditions according to Silanikove and Koluman (2015). Under heat stress, increases in RR and HR represent important thermoregulatory mechanisms that facilitate heat dissipation and maintenance of homeothermy (Barbosa et al. 2024).

Despite the increases in RR and HR, RT remained within the physiological range for sheep, suggesting that these thermoregulatory mechanisms were effective in maintaining internal body temperature stability. Similar findings have been reported in sheep exposed to tropical environmental conditions, in which respiratory and cardiovascular adjustments contributed to preserving homeothermy even under elevated thermal load (Castro et al. 2020; Façanha et al. 2021).

Lambs fed the SCA diet showed greater surface and infrared temperatures in several body regions than animals fed the SCA + TD diet. Although the SCA + TD diet presented higher NDF and ADF concentrations, the lower body temperatures observed in these animals cannot be explained solely by dietary fiber concentration. Differences in the physicochemical characteristics of the fiber, fermentation kinetics, crude protein content, and the presence of secondary metabolites in Tithonia diversifolia may have influenced ruminal fermentation and the heat increment associated with digestion. Because digestibility, fermentation end-products, and metabolic heat production were not measured in the present study, these mechanisms remain speculative. Therefore, the observed physiological responses should be interpreted as associations with dietary composition rather than evidence of a specific causal mechanism (Nobre et al. 2016).

Greater temperatures observed in the flank region, anatomically associated with the rumen, reinforce the relationship between ruminal fermentation and body heat production. Ruminal fermentation is an exothermic process that contributes substantially to metabolic heat generation in ruminants (Montanholi et al. 2008; Barbosa et al. 2024). Therefore, the lower surface and infrared temperatures observed in lambs fed SCA + TD may indicate improved thermal balance under the environmental conditions evaluated in this study. However, because animal performance, digestibility, metabolic heat production, and welfare indicators were not evaluated, these results should be interpreted as evidence of altered physiological responses rather than definitive proof of improved thermoregulatory capacity.

No significant differences were observed in enteric methane production between dietary treatments. Although *T. diversifolia* contains secondary metabolites that have been reported to influence ruminal fermentation in other studies, the inclusion level evaluated in the present study did not alter methane emissions (Rivera et al. 2022). Therefore, under the experimental conditions adopted, the inclusion of *T. diversifolia* cannot be considered an effective methane-mitigation strategy. These findings indicate that the primary effects of the diet were associated with physiological responses rather than changes in enteric methane production.

Dietary treatment affected the abundance of *Ruminococcus flavefaciens*, which was greater in lambs fed SCA + TD. This microorganism is recognized as one of the major fibrolytic bacteria in the rumen and plays an important role in the degradation of structural carbohydrates, particularly cellulose and hemicellulose (Kozloski 2011). The greater abundance of *Ruminococcus flavefaciens* may reflect an adaptive response of this fibrolytic bacterial population to the characteristics of the SCA + TD diet. Because only selected ruminal microbial populations were quantified by qPCR, these results should not be interpreted as evidence of broader changes in the ruminal microbiome. In contrast, no significant effects were observed for the remaining microbial populations evaluated, including methanogenic microorganisms. These findings may partially explain the absence of differences in enteric CH_4_ production between treatments. *Selenomonas ruminantium* populations were not affected by dietary treatment, possibly due to the concentrate proportion used in both diets. This microorganism is associated with propionate metabolism and lactate utilization and is commonly stimulated by diets containing readily fermentable carbohydrates (Shinkai et al. 2024).

## Conclusion

The inclusion of *T. diversifolia* in sugarcane silage was associated with lower respiratory rate and reduced surface and infrared body temperatures, suggesting improved thermal balance under the experimental conditions. In addition, the SCA + TD diet increased the abundance of *Ruminococcus flavefaciens*, whereas the other selected ruminal microbial populations quantified by qPCR were not affected.

However, the inclusion of *T. diversifolia* did not affect enteric CH_4_ production under the conditions evaluated in this study. These findings suggest that the use of *T. diversifolia* may contribute to improved thermal balance in lambs raised under tropical conditions, although additional nutritional strategies may be necessary to effectively mitigate enteric methane emissions.

## Data Availability

The data that support this study will be shared upon reasonable request to the corresponding author.
